# PP46 Economic Value Of Minimally Invasive Procedures: Examining Revolutionary And Evolutionary Devices In Aortic Stenosis And Benign Prostatic Hyperplasia

**DOI:** 10.1017/S0266462324002174

**Published:** 2025-01-07

**Authors:** Schezn Lim, Georgia Swan, Jennifer Sara Evans, Andrew Lim, Natsumi Fujita, Justin Lo, Virginia Priest

## Abstract

**Introduction:**

Minimally invasive procedures (MIPs) shorten procedural, hospital, and patient recovery time, providing patient benefits and reducing healthcare resource use (HCRU). This study explored the economic impact of two MIPs as discussed in published literature: transcatheter aortic valve replacement (TAVI), a step-change in cardiac clinical practice (“revolutionary”); and water vapor thermal therapy (WVTT), a progressive innovation in benign prostatic hyperplasia (BPH) (“evolutionary”).

**Methods:**

Two pragmatic literature reviews were conducted to identify studies reporting comparative HCRU for TAVI versus surgical aortic valve replacement (SAVR) in aortic stenosis (AS), and WVTT versus other procedures in BPH. Searches were conducted on 20 October 2023 in the MEDLINE and MEDLINE plus Embase databases for the AS and BPH reviews, respectively. Studies from Asia-Pacific (AS review) and all geographies (BPH review) were included. HCRU data reported in the literature were identified and extracted.

**Results:**

Forty eligible studies were included (14 AS, 26 BPH). Commonly reported outcomes were hospital length of stay (LOS) (AS, BPH studies), intensive care unit (ICU) LOS (AS studies), and procedure duration (BPH studies). In AS studies, hospital LOS was shorter for TAVI (8.00 to 19.96 days) than SAVR (13.09 to 29.50 days). ICU LOS was shorter with TAVI (0.00 to 6.40 days) than SAVR (1.00 to 8.13 days). In BPH, WVTT had shorter hospital LOS (0.00 to 1.10 days) than other procedures (1.00 to 3.00 days). Of five studies reporting procedure duration, four showed shorter procedure time with WVTT (4.00 to 30.00 mins) than other procedures (5.20 to 148.00 mins).

**Conclusions:**

MIPs, whether “revolutionary” or “evolutionary,” could offer notable time-savings (both in terms of procedure duration and length of stay) at the hospital level, which may also lead to cost savings. This highlights the importance of establishing mechanisms in payer-level economic evaluations or health technology assessments that can account for the time-saving advantages of MIPs at the hospital level.

